# Greater Dietary Inflammatory Potential Is Associated With Higher Likelihood of Abdominal Aortic Calcification

**DOI:** 10.3389/fcvm.2021.720834

**Published:** 2021-08-13

**Authors:** Zheng Qin, Kaixi Chang, Ruoxi Liao, Luojia Jiang, Qinbo Yang, Baihai Su

**Affiliations:** ^1^Department of Nephrology, National Clinical Research Center for Geriatrics, West China Hospital, Sichuan University, Chengdu, China; ^2^Med-X Center for Materials, Sichuan University, Chengdu, China

**Keywords:** dietary inflammatory index, abdominal aortic calcification, NHANES, cross-sectional study, likelihood

## Abstract

**Aims:** We aimed to assess the association between dietary inflammation index (DII) and abdominal aortic calcification (AAC) in US adults aged ≥40 years.

**Methods:** Data were obtained from the 2013–2014 National Health and Nutrition Examination Survey (NHANES). Participants who were <40 years old and missing the data of DII and AAC were excluded. DII was calculated based on a 24-h dietary recall interview for each participant. AAC score was quantified by assessing lateral spine images and severe AAC was defined as AAC score >6. Weighted multivariable regression analysis and subgroup analysis were preformed to estimate the independent relationship between DII with AAC score and severe AAC.

**Results:** A total of 2,897 participants were included with the mean DII of −0.17 ± 2.80 and the mean AAC score of 1.462 ± 3.290. The prevalence of severe AAC was 7.68% overall, and participants in higher DII quartile tended to have higher rates of severe AAC (Quartile 1: 5.03%, Quartile 2: 7.44%, Quartile 3: 8.38%, Quartile 4: 10.46%, *p* = 0.0016). A positive association between DII and AAC score was observed (β = 0.055, 95% CI: 0.010, 0.101, *p* = 0.01649), and higher DII was associated with an increased risk of severe AAC (OR = 1.067, 95% CI: 1.004, 1.134, *p* = 0.03746). Subgroup analysis indicated that this positive association between DII and AAC was similar in population with differences in gender, age, BMI, hypertension status, and diabetes status and could be appropriate for different population settings.

**Conclusion:** Higher pro-inflammatory diet was associated with higher AAC score and increased risk of severe AAC. Anti-inflammatory dietary management maybe beneficial to reduce the risk of AAC.

## Introduction

Vascular calcification (VC) is a pathology characterized by ectopic calcification in the vessel wall of muscular or elastic arteries (1), which can be commonly observed in patients with diabetes, chronic kidney disease (CKD), hypertension, osteoporosis, etc. (2–4). It contributes to a series of cardiovascular diseases (CVDs) including atherosclerosis, hypertension, aortic stenosis, and coronary heart disease and associated with a poor prognosis including higher risk of mortality, adverse cardiovascular events (stroke, acute myocardial infarction, peripheral vascular diseases, etc.), and other comorbidities (5). Meanwhile, severe calcification poses a great challenge to the treatment. There is currently no validated effective treatment for VC, and the underlying mechanisms have not been fully elucidated. Sodium thiosulfate has been reported as a potential treatment method, but more studies are still necessary (6). Djuric et al. found that sodium thiosulfate positively affected calcification progress in iliac arteries and heart valves but failed to hinder the calcification progress of abdominal aorta in a small randomized controlled trial (7). Further studies are still needed to validate the potential clinical application. After the onset of CVDs accompanied with VC, it is difficult to reverse the diseased vessels. Additionally, VC itself can hinder its treatment. For example, coronary artery disease complicated with severe calcification was still a problem in percutaneous coronary intervention treatment (8). Both clinical and epidemiological data showed that VC was closely related to higher cardiovascular disease risk and mortality, thus, the prevention and management of VC is of great significance (9, 10).

The abdominal aorta is a site prone to atherosclerosis and calcification, which could act as a good indicator of VC in patients and predicted both cardiovascular and all-cause mortality (11–13). It has been reported that the prevalence of abdominal aortic calcification (AAC) increased with age, ranging from 60% at age 65–69 years to 96% at 85 years and older. In addition, patients with AAC showed an increased risk of cardiovascular death and all-cause mortality than those without AAC (14). To evaluate the severity of calcified vessels, Kauppila et al. described an AAC grading quantification method (AAC score) by using lateral lumbar radiography in a Framingham study subgroup and found it could predict all-cause mortality and non-fatal cardiovascular events in hemodialysis patients independently (15, 16). Recently, the development of imaging technology helps us to understand VC more comprehensively. In 2009, the Kidney Disease: Improving Global Outcomes (KDIGO) suggested that abdominal lateral X-ray film should be used instead of CT as the basic imaging examination to evaluate whether abdominal aorta calcification exists and suggested that AAC score could reflect aortic calcification in peritoneal dialysis patients (17).

As a potential target for therapeutic measures, the management of diet is the most basic treatment in clinical practice and the dietary influence on health has aroused wide attention. Shivappa et al. developed the dietary inflammatory index (DII), a literature-derived, population-based dietary score summarizing the effect of dietary parameters on inflammation, which categorizes an individual's diet into proinflammatory and anti-inflammatory diet (18). A positive higher DII score means a more proinflammatory effect and a negative lower DII score indicates a more anti-inflammatory effect. Previous studies have documented a direct association between DII and a higher risk for cancer, metabolic syndrome, sarcopenia, etc. (19, 20). Li et al. reported that dietary patterns with a higher proinflammatory potential were associated with higher risk of coronary heart disease and strokes, and reducing the dietary inflammatory potential may potentially provide an effective strategy for CVD prevention (21).

Although the mechanisms involved in VC are complex and related to multiple factors, current studies have mainly found that inflammation can promote VC (22). However, the relationship between dietary inflammatory potential and abdominal aortic calcification has not been reported before. Using data from the National Health and Nutrition Examination Survey (NHANES), the aim of this study was to explore the association of dietary inflammatory potential and AAC, which may shed new light on the management of VC in clinical practice.

## Materials and Methods

### Study Population

We obtained data from the NHANES, which is a cross-sectional study to evaluate the nutrition and health status of non-institutionalized population in the USA on a repeated 2-year cycle conducted by the National Center for Health Statistics (NCHS) (23). All NHANES data are publicly available at https://www.cdc.gov/nchs/nhanes/.

**Figure 1:**
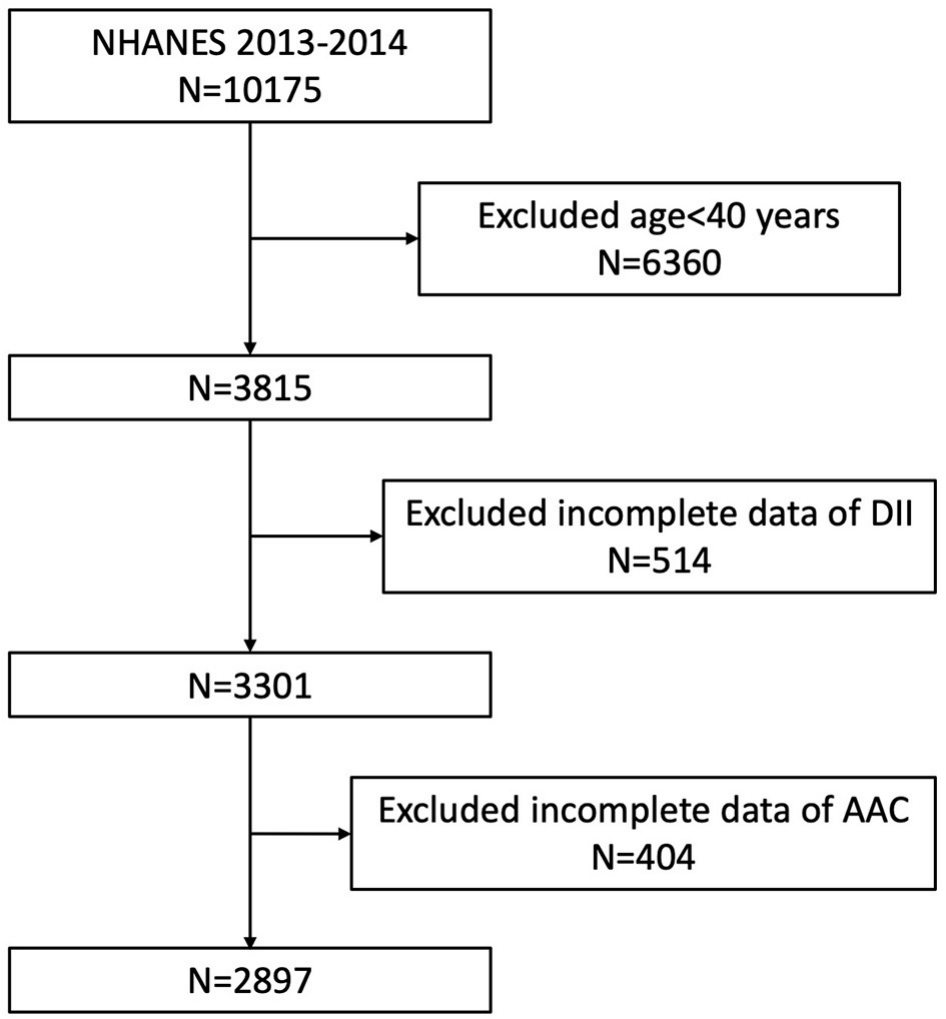
Flowchart of the sample selection from NHANES 2013–2014.

Our study was based on the data from NHANES 2013 to 2014, since only this cycle included both data on abdominal artery calcification and dietary inflammatory index. Because the data about AAC score was not available for participants aged <40 years (they were excluded for the DXA scan in NHANES 2013–2014), we enrolled participants aged ≥40 years with complete data both on AAC score and DII in our analysis. A total of 10,175 individuals were enrolled at first, after exclusion of participants aged <40 years old (*n* = 6,360), missing the dietary data relating to DII (*n* = 514), or missing the data of AAC (*n* = 404), 2,897 subjects aged ≥40 years were included in our final analysis (Figure 1).

### Exposure and Outcome Definitions

DII score was designed as exposure variable. It was designed to evaluate the inflammatory potential of diets based on the proinflammatory and anti-inflammatory properties of the dietary components (18). Dietary data in NHANES were obtained by a 24-h dietary recall interview and have been validated by the Nutrition Methodology Working Group (24). We calculated the DII based on the 24-h dietary recall data and used this to assess the dietary inflammatory potential for each individual. A positive higher DII suggested a proinflammatory effect and negative lower DII suggested an anti-inflammatory effect of diet (18). Twenty-eight food parameters were available in NHANES 2013–2014 and were used to calculate DII, namely, alcohol, β-carotene, cholesterol, carbohydrates, energy, fats, fibers, folic acid, iron, magnesium, zinc, selenium, vitamin A, vitamin B-6, vitamin B-12, vitamin C, vitamin D, vitamin E, mono-unsaturated fatty acid, protein, niacin, riboflavin, n-3 fatty acid, n-6 fatty acid, poly-unsaturated fatty acid, saturated fat, caffeine, and thiamin. Inflammatory effect scores for each dietary parameter were calculated, and the sum of the scores of each food parameter for each individual was the DII score. It has been proven that using food parameters <30 to calculate DII would not reduce its predictive ability (25, 26). DII was analyzed as a continuous variable, and participants were divided to quartiles based on the DII score for further analysis.

AAC score and severe AAC were designed as outcome variables. AAC score was used to evaluate the severity of calcified vessels, and a higher AAC score indicated a more severe vascular calcification condition. It was quantified according to the Kauppila score system by assessing lateral lumbar spine images (vertebrae L1 to L4) obtained using dual-energy X-ray absorptiometry (DXA, Densitometer Discovery A, Hologic, Marlborough, MA, USA) (16). In the Kauppila scoring method for AAC, both the anterior and posterior aortic walls were divided into four segments corresponding to the region in front of the lumbar vertebrae L1 to L4. In these four segments, aortic calcification is visually either a diffuse white spot of the aorta extending to the anterior and/or posterior aortic walls or a white linear calcification of the anterior and/or posterior aortic walls. Different calcification severity of each segment corresponded to different scores according to the calcific deposit proportions, and the sum of scores for all segments was the final AAC score, resulting in a range from “0 to 6” for each segment and “0 to 24” for the total score (16). According to previous studies, we defined severe AAC as a total AAC score >6, which was considered significant aortic calcification and has been widely used as a cutoff point in previous studies (16, 27, 28). In NHANES 2013–2014, participants who were aged <40 years old, pregnant, self-reported history of radiographic contrast material (barium) use in the past 7 days, weighted over 450 lbs., and a Harrington Rod in the spine for scoliosis were excluded for DXA scans, and their AAC score data were not available.

### Covariates

Continuous variables in our study included age (year), body mass index (BMI, kg/m^2^), systolic blood pressure (SBP, mmHg), diastolic blood pressure (DBP, mmHg), and serum creatinine (mg/dl). Categorical variables included gender, race, education level, ratio of family income to poverty (RIP), hypertension, diabetes, congestive heart failure (CHF), and smoke. All detailed measurement process of these variables was publicly available at www.cdc.gov/nchs/nhanes/.

### Statistical Analysis

Statistical analyses were conducted according to CDC guidelines (29). All analysis was performed using appropriate NHANES sampling weights and accounted for complex multistage cluster survey design. Continuous variables were presented as mean with standard deviation, and categorical variables were presented as frequency or percentage. Weighted Student's *t*-test (for continuous variables) or weighted chi-square test (for categorical variables) was employed to assess the differences in groups divided by DII (quartiles). Weighted multiple regression analysis was preformed to estimate the independent relationship between DII scores and AAC (including AAC score and severe AAC) in three different models. In model 1, no covariates were adjusted. Model 2 was adjusted for gender, age, and race. Model 3 was adjusted for gender, age, race, education level, PIR, SBP, DBP, BMI, serum creatinine, hypertension, diabetes, congestive heart failure, and smoke. Subgroup analysis was performed by stratified multivariate regression analysis. In addition, weighted generalized additive models and smooth curve fittings were used to address the non-linearity of DII and AAC. *p* < 0.05 was considered statistically significant. All analysis was preformed using Empower software (www. empowerstats.com; X&Y solutions, Inc., Boston MA) and R version 3.4.3 (http://www.R-project.org, The R Foundation).

## Results

### Baseline Characteristics of Participants

The weighted demographic characteristics and other covariates of included participants are shown in Table 1. A total of 2,897 participants were included in our study, of whom 47.58% were males and 52.42% were females with an average age of 57.47 ± 11.47 years old. The mean of DII was −0.17 ± 2.80, and the ranges of DII for quartiles 1–4 were −6.94 to −2.27, −2.27 to −0.30, −0.30 to 1.85, and 1.85 to 6.89, respectively. Among different quartiles of DII, significant differences were observed in gender, race, education level, PIR, SBP, BMI, whether having hypertension, congestive heart failure, AAC score, and the prevalence of severe AAC, while the difference between quartiles in DBP, serum creatinine, diabetes, and smoke status did not meet the statistical significance. The average AAC score was 1.462 ± 3.290 for the whole participants, and the mean AAC score was 1.811 ± 3.884 for the highest quartile and 1.049 ± 2.806 for the lowest quartile. The prevalence of severe AAC was 7.68% overall, and participants in higher DII quartile tended to have higher rates of severe AAC (Quartile 1: 5.03%, Quartile 2: 7.44%, Quartile 3: 8.38%, Quartile 4: 10.46%, *p* = 0.0016).

**Table 1:** Baseline characteristics of participants, weighted.

	**Overall** **(−0.17 ± 2.80)**	**Quartile 1** **(−6.94 to −2.27)**	**Quartile 2** **(−2.27 to −0.30)**	**Quartile 3** **(−0.30 to 1.85)**	**Quartile 4** **(1.85–6.89)**	***p*** **-value**
Age (year)	57.47 ± 11.47	56.14 ± 11.19	57.99 ± 11.32	57.46 ± 11.37	58.50 ± 11.94	<0.0001
**Gender (%)**						
Male	47.58	67.26	49.40	37.90	31.66	<0.0001
Female	52.42	32.74	50.60	62.10	68.34	
**Race (%)**						
Mexican American	6.74	7.21	7.80	6.52	5.14	<0.0001
Other hispanic	4.62	4.07	4.55	5.79	4.11	
Non-hispanic white	71.92	74.70	73.52	70.00	68.65	
Non-hispanic black	9.99	7.62	8.44	10.01	14.75	
Other races	6.73	6.40	5.70	7.68	7.35	
**Education level (%)**						
Less than high school	14.74	12.14	12.34	15.47	20.05	<0.0001
High school or GED	21.52	16.21	23.13	22.34	25.21	
Above high school	63.73	71.66	64.53	62.19	54.72	
Unknown	0.01	0.00	0.00	0.00	0.03	
**RIP**						
<1	11.48	9.84	7.44	12.77	16.87	<0.0001
≥1	88.52	90.16	92.56	87.23	83.13	
SBP (mmHg)	125.17 ± 17.76	123.52 ± 16.60	124.54 ± 17.44	125.92 ± 18.05	127.16 ± 18.93	0.0012
DBP (mmHg)	71.22 ± 12.17	71.05 ± 11.70	71.48 ± 12.17	71.20 ± 11.93	71.12 ± 12.95	0.9169
BMI (kg/m2)	28.59 ± 5.54	28.20 ± 5.53	28.67 ± 5.48	28.51 ± 5.58	29.05 ± 5.54	0.0369
Creatinine (mg/dl)	0.92 ± 0.41	0.94 ± 0.27	0.94 ± 0.34	0.89 ± 0.53	0.92 ± 0.47	0.0675
Hypertension (%)	55.90	36.04	44.07	45.26	52.75	<0.0001
Diabetes (%)	87.11	9.93	12.11	11.98	18.43	0.2430
CHF (%)	2.69	1.11	3.56	2.54	3.74	0.0057
Smoke (%)	45.35	47.15	43.44	44.11	46.79	0.3714
AAC score	1.46 ± 3.29	1.05 ± 2.81	1.47 ± 3.08	1.60 ± 3.37	1.81 ± 3.88	0.0001
Severe AAC (%)	7.68	5.03	7.44	8.38	10.46	0.0016

*GED, general educational development; RIP, ratio of family income to poverty; SBP, systolic blood pressure; DBP, diastolic blood pressure; BMI, body mass index; CHF, congestive heart failure; AAC, abdominal aortic calcification*.

### Higher DII Was Associated With Higher AAC Score and Higher Risk of Severe AAC

We employed weighted multiple regression analysis to assess the association between DII and AAC score in three different models. In model 1, no covariates were adjusted. Model 2 was adjusted for gender, age, and race. Model 3 was adjusted for gender, age, race, education level, PIR, SBP, DBP, BMI, serum creatinine, hypertension, diabetes, congestive heart failure, and smoke. A positive association between DII and AAC was observed both in model 1 (β = 0.088, 95% CI: 0.044, 0.131, *p* = 0.00008) and model 2 (β = 0.062, 95% CI: 0.020, 0.103, *p* = 0.00383). In fully adjusted model (model 3), the positive association was still stable (β = 0.055, 95% CI: 0.010, 0.101, *p* = 0.01649), indicating that each unit of increased DII score was associated with 0.055 increased unit of AAC score, respectively. We also converted DII from a continuous variable to a categorical variable (quartiles) to conduct the sensitivity analysis. Compared with the lowest DII scores quartile (Quartile 1), the AAC score increased with the higher DII quartiles group. The mean AAC score of the highest DII quartile (Quartile 4) was 0.517 unit higher compared with the lowest quartile (β = 0.517, 95% CI: 0.168, 0.866; *p* = 0.00374), respectively (Table 2).

**Table 2:** Higher DII associated with higher AAC score and higher risk of severe AAC.

	***β*****/OR (95% CI)**, ***p*****-value**		
	**Model 1** ^***a***^	**Model 2** ^***b***^	**Model 3** ^***c***^
**AAC score**			
DII	0.088 (0.044, 0.131) 0.00008	0.062 (0.020, 0.103) 0.00383	0.055 (0.010, 0.101) 0.01649
**DII categories**			
Quartile 1	Reference	Reference	Reference
Quartile 2	0.418 (0.093, 0.743) 0.01178	0.219 (−0.083, 0.520) 0.15585	0.272 (−0.049, 0.594) 0.09654
Quartile 3	0.554 (0.219, 0.889) 0.00122	0.419 (0.104, 0.734) 0.00928	0.347 (0.014, 0.679) 0.04095
Quartile 4	0.762 (0.421, 1.104) 0.00001	0.522 (0.196, 0.848) 0.00174	0.517 (0.168, 0.866) 0.00374
**Severe AAC** ^***d***^			
DII	1.090 (1.041, 1.140) 0.00020	1.066 (1.012, 1.124) 0.01625	1.067 (1.004, 1.134) 0.03746
**DII categories**			
Quartile 1	Reference	Reference	Reference
Quartile 2	1.589 (1.066, 2.367) 0.02292	1.379 (0.895, 2.126) 0.14499	1.432 (0.886, 2.315) 0.14270
Quartile 3	1.695 (1.142, 2.515) 0.00876	1.553 (1.010, 2.389) 0.04514	1.521 (0.941, 2.458) 0.08686
Quartile 4	2.075 (1.415, 3.044) 0.00019	1.663 (1.084, 2.552) 0.01979	1.679 (1.034, 2.726) 0.03599

*In sensitivity analysis, dietary inflammatory index was converted from a continuous variable to a categorical variable (quartiles)*.

*β, effect sizes; OR, odds ratio; 95% CI, 95% confidence interval; DII, dietary inflammatory index; AAC, abdominal aortic calcification*.

a *No covariates were adjusted*.

b *Adjusted for gender, age, and race*.

c *Adjusted for gender, age, race, education level, ratio of family income to poverty, systolic blood pressure, diastolic blood pressure, body mass index, creatinine, hypertension, diabetes, congestive heart failure, and smoke*.

d *Severe AAC was defined as AAC score >6*.

As for severe AAC, we also observed that increased DII was associated with a higher risk of severe AAC (model 1, OR = 1.090, 95% CI: 1.041, 1.140, *p* = 0.00020; model 2, OR = 1.066, 95% CI: 1.012, 1.124, *p* = 0.01625; model 3, OR = 1.067, 95% CI: 1.004, 1.134, *p* = 0.03746). In model 3, which was adjusted for all covariates, our results indicated that each unit of increased DII score was associated with 6.7% increased risk of severe AAC. In sensitivity analysis, the adjusted OR (reference to Quartile 1) was 1.679 (95% CI: 1.034, 2.726, *p* = 0.03599) for Quartile 4, suggesting a stable positive relationship between increased DII and increased risk of severe AAC with statistical significance (Table 2).

**Figure 2:**
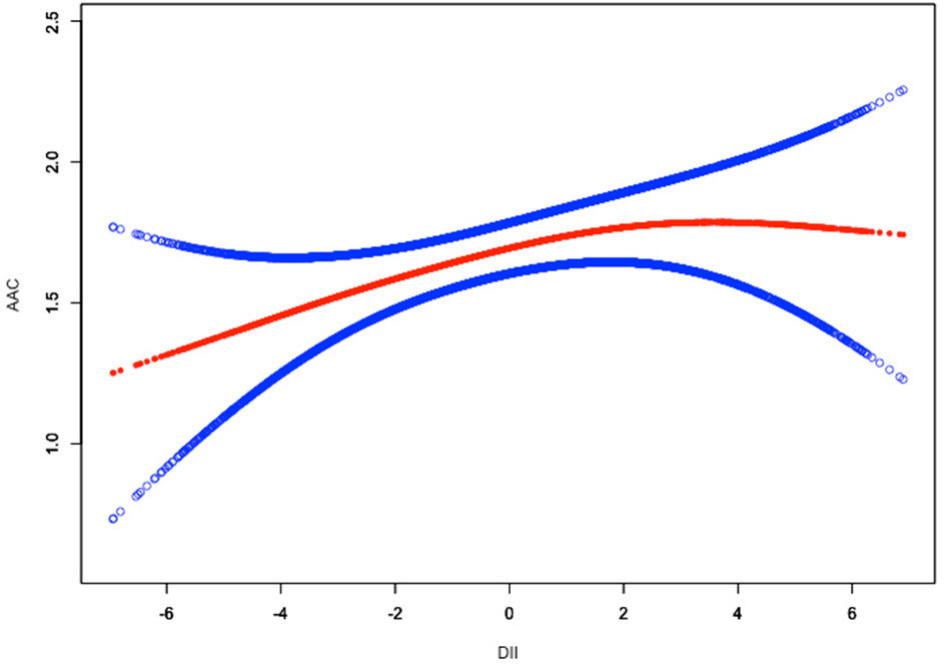
Linear relationship between DII and AAC score by the generalized additive model.

**Figure 3:**
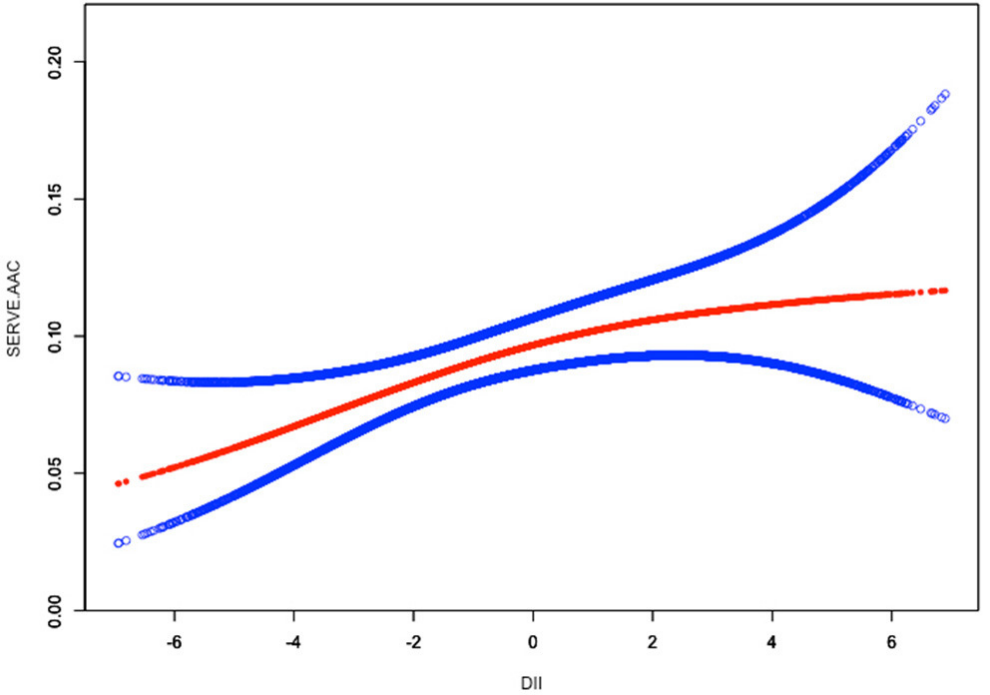
Linear relationship between DII and severe AAC by the generalized additive model.

To assess the non-linear relationship between DII and ACC, weighted generalized additive models and smooth curve fittings were employed for further study. The results indicated that there was no non-linear relationship between DII and AAC score (Figure 2). A similar result was observed in the association between DII and severe AAC (Figure 3).

### DII Positively Associated With Higher AAC Score in Different Subgroups

Subgroup analysis was conducted to further explore the association between DII and AAC score in different population setting including gender, age, BMI, hypertension, and diabetes (Table 3). We also performed an interaction test to evaluate if there was any significant dependence of the effect modifier on this association. *p* for interaction >0.05 means no significant dependence was observed. In our analysis, no correlation with the *p* for interaction meeting the statistical significance was detected (all *p* for interaction >0.05), indicating that the magnitude of the association was the same for different population settings.

**Table 3:** Subgroup analysis.

**DII**	**AAC score**		**Severe AAC**	
	**β (95% CI), *p* for trend**	***p*** **for interaction**	**OR (95% CI)**, ***p*****for trend**	***p*** **for interaction**
**Subgroup analysis stratified by gender**				
Male	0.038 (0.024, 0.086) 0.04872	0.2645	1.059 (1.045, 1.139) 0.01480	0.3986
Female	0.087 (0.021, 0.153) 0.01016		1.088 (1.006, 1.189) 0.01135	
**Subgroup analysis stratified by age (years)**				
Age <60	0.021 (0.012, 0.053) 0.01570	0.2534	1.043 (1.029, 1.207) 0.00852	0.5528
Age ≥60	0.118 (0.019, 0.217) 0.02026		1.085 (1.016, 1.158) 0.01500	
**Subgroup analysis stratified by BMI (kg/m^2^)**				
BMI ≤ 30	0.083 (0.003, 0.166) 0.04966	0.5308	1.084 (1.066, 1.216) 0.02003	0.7264
BMI >30	0.068 (0.002, 0.138) 0.03585		1.016 (1.049, 1.141) 0.01836	
**Subgroup analysis stratified by hypertension**				
Yes	0.024 (0.057, 0.105) 0.02650	0.4856	1.064 (1.008, 1.145) 0.02955	0.3399
No	0.055 (0.008, 0.101) 0.02076		1.067 (1.003, 1.194) 0.02144	
**Subgroup analysis stratified by diabetes**				
Yes	0.022 (0.014, 0.130) 0.02547	0.3482	1.021 (1.022, 1.112) 0.03028	0.1189
No	0.057 (0.011, 0.103) 0.01516		1.092 (1.014, 1.177) 0.02044	

*The results of subgroup analysis were adjusted for all covariates except effect modifier*.

*β, effect sizes; 95% CI, 95% confidence interval; OR, odds ratio*.

Regarding the correlation between DII and AAC score, in subgroups stratified by gender, age, BMI, hypertension, and diabetes, the positive associations between DII and AAC score in different subgroups were all significant (*p* for trend < 0.05) and *p* for interaction >0.05, suggesting that the correlation between DII scores and AAC score was similar in population with differences in gender, age, BMI, hypertension status, and diabetes status.

### DII Positively Associated With Increased Risk of Severe AAC in Different Subgroups

As for the association between DII and severe ACC, the results of subgroup stratified by gender demonstrated that DII was positively associated with higher risk of severe AAC both in female subgroup (OR = 1.088, *p* for trend = 0.01135) and male subgroup (OR = 1.059, *p* for trend = 0.01480) with *p* for interaction of 0.3986, indicating that there was no significant dependence of gender on this positive association between DII and severe AAC.

Similarly, we did not find any significant dependence on age, BMI, hypertension, diabetes status (all *p* for interaction >0.05). These results indicated that the positive association between DII and the risk of severe AAC was similar in population with differences in gender, age, BMI, hypertension status, and diabetes status and could be appropriate for various population settings as well (Table 3).

## Discussion

In this cross-sectional study including 2,897 adults, a significant positive association between DII with AAC score and severe ACC was observed, indicating that a proinflammatory diet may contribute to higher AAC score and increased risk of severe AAC. The associations between the exposure variable and outcomes were still stable in subgroups stratified by gender, age, BMI, hypertension status, and diabetes status, suggesting that this positive association may be appropriate for different population settings.

To our knowledge, this is the first study assessing the association between the dietary inflammatory potential and AAC. Previous studies have revealed the influence of various dietary factors on calcification. Experimental studies have shown the roles of high phosphorus and calcium diets as promoters of calcification, and magnesium and vitamin K intakes as inhibitory factors (30–34). Obesity and metabolic syndrome could also promote VC, and vitamin E can protect against calcification in uremic rats (35–37). In addition, Lu et al. reported that high serum selenium concentration was positively associated with a mean AAC score and an increased risk of severe AAC among non-institutionalized US adults independently (38).

As one of the most appropriate approaches to understand the relationship between diet and the risk of various diseases, dietary pattern analysis has been regarded as a more robust and valid measure of dietary intake in the longer term (39). It has been demonstrated that a proinflammatory diet can increase systemic inflammation (40). For example, the “Western” diet containing high intake in red meat, high-fat dairy products, refined grains, and simple carbohydrates, has been reported to be associated with higher levels of CRP and interleukin (IL)-6 (41, 42). In contrast, the Dietary Approaches to Stop Hypertension (DASH) diet emphasized intake of fruits, vegetables, low fat dairy foods, and reduced saturated and total fat, which can suppress the inflammation process (43). Likewise, the Mediterranean diet pattern, which is high in fruit, vegetables, and healthy fats, has been reported to be associated with reduced concentrations of CRP (44). Recently, DII has been gradually explored by more scholars as a variable risk factor on health and disease in a number of specialized areas, including cardiovascular disease, metabolic syndrome (19, 45), cancer (46–48), obstetrics (49), digestive system disease (50, 51), etc. Relevant to our study, inflammation was regarded to play a crucial role in VC.

Traditionally, VC was classified into two forms, depending on the place where the mineral deposit is located. Intimal calcification was closely related to lipid deposits, and the clinically relevant infiltration of inflammatory cells, with obstructive arterial disease (34), and medial calcification was associated with aging, diabetes mellitus, hypertension, osteoporosis, and CKD (22). It is recognized as a cell-mediated, active process involving a series of related genes and proteins, with an imbalance of promoters and inhibitors. With the advances in the field of VC pathogenesis, our current understanding of the mechanism of VC included chronic inflammation, autophagy defects, endoplasmic reticulum stress, mitochondrial dysfunction, and its dynamics (52). The exact mechanism of the positive association between DII and AAC in our study still remains unclear. A possible explanation to support our results might be the effect of diet on proinflammatory markers such as IL-1, IL-6, and tumor necrosis factor-α (TNF-α) (18, 53). Proinflammatory diet could increase the level of inflammation markers, and the increased inflammation level promotes VC (22). Previous studies have indicated that IL-1β (54), IL-6 (55), and TNF-α (56) could stimulate vascular smooth muscle cell (VSMC) calcification through various pathways. The increased IL-1β release could modulate proinflammatory signaling cascades in VSMCs to promote calcification through NF-kappa B pathway, WNT/β-catenin pathway, etc. (57, 58). IL-6 could also get involved in the VSMC calcification by inducing oxidative stress and activating RANKL, STAT3, BMP-2-WNT/β-catenin pathway, etc. (59–62). TNF-α mediated calcification *via* the upregulation of BMP-2 signaling and triggered the osteo-/chondrogenic transformation of VSMCs with an interplay with MSX-2 up-expression, WNT/β-catenin pathway, and oxidative stress (63–65). Proinflammatory macrophages also have been reported to further play a key role in VC progression (66). It could be inferred that proinflammatory diet may increase the inflammation level, and the continuously activated inflammatory state could lead to the activation of inflammation-related signaling pathways, macrophage infiltration, and T-lymphocyte activation, which in turn activated the differentiation of VSMCs into osteoblastic phenotype and calcium salt deposition, resulting in the progress of vascular calcification.

One of the strengths of our study is that it was based on a nationwide, population-based sampling survey data and appropriate weighting of survey participants was adopted, making the findings widely usable in the US population. In addition, considering the relationship of ACC with some other risk factors, several covariables related to AAC including gender, age, BMI, serum creatinine, hypertension, diabetes, congestive heart failure, smoke, etc. were included in the adjusted model for weighted multiple regression analysis (67–69). Moreover, our large sample size allowed us to perform subgroup analyses. Our results indicated that the positive association between DII and AAC was similar in population with differences in gender, age, BMI, hypertension status, and diabetes status. Given the fact that aging was originally one of the strongest drivers of VC in the elderly, the prevalence of AAC was expected to be increased among older population (70). For the subgroup analysis stratified by age, we found that DII was positively associated with higher AAC score (β = 0.021, *p* for trend = 0.01570) and higher risk of severe AAC (OR = 1.043, *p* for trend = 0.00852) in the <60 year-old subgroup. In the ≥60-year-old group, we observed a similar result (for AAC score, β = 0.118, *p* for trend = 0.02026; for severe AAC, OR = 1.085, *p* for trend = 0.01500). Interaction test indicated that the magnitude of the association was the same for population with different ages (for AAC score, *p* for interaction = 0.2534; for severe AAC, *p* for interaction = 0.5528). Indeed, for each unit of increased DII, a 4.3% and 8.5% increased risk of severe AAC was observed for participants aged <60 and ≥60 years, which was consistent with the fact that older population showed a higher AAC prevalence and had higher risks of AAC.

The limitations of this study cannot be ignored. Firstly, since NHANES was a cross-sectional survey in US population, we could not obtain a causal relationship between dietary inflammatory potential with AAC, and the ability of our findings to be generalized to a common population or other ethnic groups may be limited. Therefore, prospective studies with a larger sample size are required to clarify the causality. Secondly, we cannot completely rule out residual confounders due to unmeasured or unknown variables. For example, even in the non-hospitalized population, there is a possibility that medication may affect VC, such as vitamin K antagonists (71) and statins (58, 72). In addition, the data on AAC scores for participants aged <40 years old was not available in NHANES 2013–2014. Thus, we could not analyze this association for wider age groups. The detailed molecular mechanisms supporting our findings are still uncertain and need more laboratory-based real experiments. Finally, the DII was calculated from a detailed and validated dietary questionnaire, but the diet was subject to change over time, thus we cannot account for dietary changes. Since the DII was calculated based on the 24-h dietary recall data, some misclassification of the exposure and recall bias were inevitable. Thus, further researches are still needed to gain insight into the long-term association between dietary inflammatory potential and AAC.

## Conclusion

In this cross-sectional study with 2,897 adults included, a significant positive association between DII and AAC was observed, indicating that higher consumption of proinflammatory diet might contribute to a higher AAC score and increased risk of severe AAC. Our finding suggests that a proper anti-inflammatory dietary management maybe beneficial to lower the risk of vascular calcification in adults. However, further research and clinical settings are still needed to validate for potential application.

## Data Availability Statement

Publicly available datasets were analyzed in this study. This data can be found here: https://www.cdc.gov/nchs/nhanes/.

## Ethics Statement

The studies involving human participants were reviewed and approved by The NCHS Ethic Review Board. The patients/participants provided their written informed consent to participate in this study.

## Author Contributions

ZQ: data analysis, software, and writing (original draft). KC: formal analysis and writing (original draft). RL: methodology and writing (original draft). LJ and QY: formal analysis and software. BS: conceptualization, funding acquisition, and writing (reviewing and editing). All authors contributed to the article and approved the submitted version.

## Conflict of Interest

The authors declare that the research was conducted in the absence of any commercial or financial relationships that could be construed as a potential conflict of interest.

## Publisher's Note

All claims expressed in this article are solely those of the authors and do not necessarily represent those of their affiliated organizations, or those of the publisher, the editors and the reviewers. Any product that may be evaluated in this article, or claim that may be made by its manufacturer, is not guaranteed or endorsed by the publisher.
